# Work-related musculoskeletal injuries amongst obstetrics and gynaecology trainees in East Midland region of the UK

**DOI:** 10.1007/s00404-017-4449-y

**Published:** 2017-07-12

**Authors:** Kubra Arslan Okuyucu, Yadava Jeve, Angie Doshani

**Affiliations:** 1 Loughborough University, Loughborough, United Kingdom; 2 University Hospitals of Leicester, Leicester, United Kingdom

**Keywords:** Work-related musculoskeletal injuries, Obstetrics, Gynaecology, Trainees

## Abstract

**Purpose:**

Work-related musculoskeletal injuries (WRMSI) have been well known amongst obstetrics and gynaecology (O&G) practitioners, but limited data have been reported. Our aim is to determine the prevalence, severity and characteristics of WRMSI amongst O&G trainees.

**Methods:**

A musculoskeletal ergonomic survey was conducted amongst the O&G trainees in the East-Midlands region of United Kingdom (UK). The survey comprised of demographic details, year of training, previous manual handling training, any work-related orthopaedic injury, the type of injury, any treatment received in addition to any sick leave incurred after the injury were also documented.

**Results:**

The response rate for the survey was 76% (59/78). The majority (22%) were senior specialist trainee, seventh year (ST7) and between 30 and 34 age groups. Approximately 90% of the trainees reported to have experienced pain in the last year. The most common site was the back, which was followed by the shoulders and the upper limbs. 63% of trainees reported injuries that were attributed to WRMSI. One in ten of the trainees needed time off work due to injury. A total of 20 days were lost in the last 12 months as a result of pain or discomfort attributed to obstetric work.

**Conclusions:**

Our results demonstrate the prevalence of work-related injuries and its detrimental effects. Such injuries are underreported on incident reporting system. Ergonomics and WRMSI prevention in obstetrics and gynaecology is an area seldom discussed. Obstetric training sessions should incorporate ergonomic interventions. Further research is required to establish relevant aetiological factors related to WRMSI in this specialty.

## Introduction

Musculoskeletal injuries (MSI) are common problems experienced by a majority of people from various occupational groups, and approximately half of the occupational injuries reported in many countries are musculoskeletal related ones [1]. According to the definition recognised by the World Health Organisation [2], work-related disorders are problems “associated with certain exposures at work, including physical and mental work-load, adverse psychosocial factors, workers’ habits and life-style, individual susceptibility, in some instance, combined occupational and environmental exposures”.

Force, which can also be called load, is required for any motion. Although there are many types of force affecting the biomechanical structure of body motion [3], they are mainly classified as internal and external forces that provide optimum movements to a human body [4]. From an ergonomics perspective, a biomechanical imbalance develops when the internal force requires greater than the capacity of person attempt to it [5]. This imbalance has a high chance of resulting in an injury. The aim of the application of ergonomics is to design the working task, system and equipment to fit the person; rather than to adapt the person to working situation [6].

It is obvious that these kinds of problems affect quality of life as well as productivity at work places. Furthermore, many studies show that they can also result in many working days lost with considerable costs [7–9]. A study requested by the UK Government estimated that absenteeism of workers in the UK cost around £15 billion a year, and £13 billion was spent for health and wellbeing services [8]. Moreover, they also added that 140 million working days were lost due to sickness absence annually.

Boorman [7] reviewed “NHS Health and Well-being in 2009 for the purpose of finding a way of improving NHS staff health and well-being. The main issues highlighted in this report were: (1) the NHS lost 10.3 million working days of sickness absence in each year; (2) sick leave for all staff in NHS cost £1.7 billion annually; and (3) approximately 50% of sickness absence was due to musculoskeletal symptoms, of which back pain was the most common one. In this report, it was also indicated that the most common reason for sickness absence was MSI, followed by stress, depression and anxiety with nearly 30% [7]. These findings indicated that health related problems, mainly MSI, have a considerable impact on both well-being and economy.

In the final part of the report, Boorman [7] made recommendations “to improve the care of staff to improve the care for patients”. The report has suggested that NHS organisations should develop strategies for prevention, with easy access to intervention services such as physiotherapy, and with life-style changes such as physical activity classes, which actively develop health and well-being. It was also stated that staff health and well-being should not only be the responsibility of occupational or well-being departments, but that each person needs to take responsibility for their own health. This review indicated that if Boorman’s recommendations were put into practice, the sickness absence would reduce, and NHS would save £555 million and obtain 3.4 million working days a year. In response to the Boorman’s recommendations, many actions and campaigns have been commenced. For example, in 2011 a guidance was realised for occupational health services to provide a better care [10]. Another example was in 2015, Nottingham University Hospitals NHS Trust started a programme to encourage the staff to be physically active [11]. Since the review was launched in 2009, there was a decrease in sickness absence of NHS workers, however, this reduction has not continued.

Many researchers have claimed that psychosocial factors including job satisfaction and stress at work are potential antecedents for the development of musculoskeletal injuries [12–14]. People having pain are also likely to report anxiety and fear symptoms. The reason for this was argued that they are worried about the impact of their symptoms on their lives and future [15]. A systematic review by Lang et al. [16] also pointed out that stress can predict severe somatic symptoms. It is, however, unclear whether psychosocial conditions have a causal role or consequence of injuries [17].

The research to date has tended to focus on nurses with manual handling related activities and resulting most commonly low back pain [12, 18, 19]. Each health profession group has different working patterns and positions; they might develop different injuries. For example, neck and upper back problems have been commonly reported in dentistry practice [20, 21]. This is likely to be because they generally work with neck bent forward and rotated, and shoulders are fixed in abduction position [20, 21]. A survey was conducted to gauge the prevalence of work-related musculoskeletal injuries (WRMSI) among professionals using ultrasound device in an international conference with 407 participants, of which the majority (*n*: 215) were obstetricians and/or gynaecologists [22]. 65.6% of all the professionals experienced injuries, and neck, back and shoulder pain were the most commonly reported complaints.

Although the studies exploring WRMSI among obstetricians are limited in the literature, they have been reported to be at a considerable risk of developing serious injuries [22–24]. These injuries may be developed by either forceful activities during labour such as pushing, pulling, and moving patients or heavy objects, or harmful and uncomfortable working postures such as repetitive tasks, working in extreme and stressful body postures, as well as static positions for a long time [6, 25].

Yoong et al. [23] conducted a cross-sectional survey study among 97 obstetrics and gynaecology (O&G) trainees in the London area. The participants were asked about their work-related orthopaedic injury, the type of injury and the impact of injury on their trainings. Twenty-eight trainees reported that they had experienced injuries due to working tasks. The participants reported injuries of shoulder and neck (*n* = 9), wrist (*n* = 7), low back (*n* = 6), forearm (*n* = 4), thumb (*n* = 3), elbow (*n* = 2), hands (*n* = 1) and ankle (*n* = 1). A total of 80 days were taken time off work due to those injuries by eight trainees. The injuries developed during caesarean sections (*n* = 8), forceps deliveries (*n* = 8), assisting at cervical cerclage (*n* = 1) and running to a delivery (*n* = 1). Parupalli et al. [24] also reported an obstetrician sustaining mallet finger deformity with rupture of distal, and inter phalangeal extensor tendon during managing a shoulder dystocia. The treatment process lasted four months.

Overall, the literature review searched so far has shown that there is a dearth of studies on the exploration of WRMSI related with obstetric work, which seems to have a noticeable impact on both health and the economy. This paper aims to determine the prevalence, severity and characteristics of WRMSI amongst O&G trainees in the East-Midlands region of UK.

## Methods

This was a cross-sectional survey study. The survey was conducted in an educational training meeting in 2015. There are three regional educational trainings run in each year in the East-Midlands region of UK. 78 obstetrics and gynaecology (O&G) trainees attended this regional training on ‘Improving your well-being’. All 78 O&G trainees attending this training session were invited to participate in the musculoskeletal ergonomic survey. The questionnaire was distributed as paper copies by the researcher. Those who were willing to participate filled out the paper copies given to them and returned them back to the researcher on the same day.

The questionnaire included 16 questions categorised into: (1) demographics; (2) injury data; (3) impact of injury; and (4) general mental health. Within the demographics, we asked about level of training, age and manual handling training. In the injury data, we explored presence of MSI during the last 12 months along with an indication of body part, severity of the injury, duration of injury and management of injury. Number of days off work due to the injury was asked to understand the impact of injury. General mental health was explored by asking questions about job satisfaction in general; anxiety and depression in the last week. Job satisfaction, anxiety, depression and severity of injury were recorded on a zero to ten scales. Data from the questionnaires were analysed on ExCEL spreadsheet. Data are reported as number (%).

## Results

Fifty-nine trainee doctors completed the questionnaires. The response rate was 76% (59 out of 78). There are different grades of trainings in the UK, which vary from specialist training levels (ST) 1–7 to post Certificate of Completion of Training (CCT), which is achieved after successful minimum seven years of training. After graduating from medical school, doctors in the UK spend two years in foundation training years working in different areas under close supervision. After the foundation training, the doctors enter into their chosen speciality. For O&G, this is a minimum seven-year program. As they progress they gain practical and analytic skills. For example, by the end of the second year (ST2), doctors in speciality training O&G would be able to carry out assisted delivery instruments such as forceps and perform an uncomplicated caesarean section. By the end of fifth year (ST5), they should be able to manage caesarean sections of undermediate complexity and work independently for gynaecological procedures. After completing the seven training years, they are awarded a CCT which allows them to work as a specialist in O&G. Trust fellows are doctors who are employed by hospitals to work at various levels depending on their experience. Participants’ year of training in this study ranged from specialist trainee, first year (ST1) to post CCT, and the majority (22%) were senior trainees (ST7). The age group category varied between 24 and 44 years, and majority (34%) of them were in 30–34 age groups (Table 1).

**Table 1:** Characteristics of participants

	Number	**%**
Age		
24–29	14	28
30–34	17	34
35–39	16	32
40–44	3	6
45–49	0	0
Year of training		
ST1	9	15
ST2	7	12
ST3	6	10
ST4	3	5
ST5	7	12
ST6	8	13
ST7	13	22
Post CCT	1	2
Trust fellow	1	2
Not stated	4	7

The results of the survey demonstrated that 88% of trainees (*n* = 50) had at least one experience of pain during the last year. Nine participants who completed the questionnaire did not report any pain or discomfort. Table 2 shows the prevalence of injuries reported by the participants. The total number of injuries was 71, with the back being the most commonly reported body part (*n* = 21), followed by the shoulder (*n* = 13) and upper limbs (*n* = 13). Around 14% of the injuries occurred all the time, while 28% were felt at least once per day. More than half of injuries (63%) were reported to be caused by work-related tasks.

**Table 2:** The prevalence of injuries in O&G trainees

Body part	Number	%
Back	21	30
Shoulder	13	18
Upper limb	13	18
Lower limb	9	13
Multiple sites	9	13
Neck	6	8

Only one trainee completed an incident form about their work-related injuries. Table 3 shows the management of the 71 injuries reported by 50 trainees. The majority of injuries (*n* = 40) were managed by a combination of physiotherapy and self-medication. The rest of them were either done nothing or seen by a doctor based in the community called General Practice (GP) or Orthopaedics. Of those 71 injuries, 48 (68%) improved, while 19 (27%) stayed the same and 3 (4.2%) worsened.

**Table 3:** Management of the 71 injuries reported by 50 trainees

Management	Number
Self-medication	31
Occupational Health/Physiotherapy	9
GP	9
Orthopaedics	1
Nothing	21

10% (*n* = 6) of the respondents needed time off work due to their injuries with the total time taken off work being 20 days in the last 12 months. Only half of the trainees (*n* = 25) had attended manual handling training in the last year, of those 19 (76%) reported that it was useful.

Majority of the trainees seemed to be satisfied with their jobs. 68% of the respondents rated a satisfaction score more than seven out of ten. In response to the question asking about their level of stress/anxiety in the last week, 29% of those who replied rated to be above seven out of ten. When the participants were asked about the level of depression, only 13% reported feeling depressed in the last seven days.

## Discussion

In reviewing the literature, very little data were found on the work-related injuries among health professionals in obstetrics. To our knowledge this current study is the second survey after Yoong et al.’s [23], which was designed to investigate the work-related injuries among O&G trainees.

The survey results of the present study showed that majority of trainees (88%) experienced MSI during the last 12 months. Back pain was the most commonly reported body part. Our results regarding the prevalence of 26% for neck and shoulder and 18% for upper limb injuries are consistent with the findings of similar previous studies [23, 26]. Neck and upper limb injuries have also been experienced very often by maternity professionals. Stichler et al. [27] identified some working tasks specific to maternity professionals that might result in MSI as: (1) maternity professionals handle pregnant women who are heavier than other patients with moving or transferring after epidural anaesthesia and positioning them. (2) They need to lean regularly to perform vaginal examination, or to listen fetal heart sounds. Another possible explanation might be that in obstetric emergencies such as shoulder dystocia, placental abruption, Caesarean section or forceps delivery, they have to adapt physically stressful positions.

The overall prevalence of WRMSI in our study was found to be 63%, this is higher than that of previously reported as 28.7% by Yoong et al. [23] among trainees in London region in 2008. As both are exploratory studies, the reason behind this difference is not clear. Some potential explanations for this may be organizational factors, work intensity or patient characteristics. With changing work schedule and shifts becoming longer for this professional group, the increased work demand has been argued to result in psychosocial impact including stress and depression [28]. Increased size and complexity of patients over the years has made the speciality more physically demanding that might result in musculoskeletal injuries in these occupational groups.

Despite the high number of injuries, only one trainee filled the incident reporting form. This highlights the fact that most of the trainees may not aware of this being a reportable incidence or may not want to repeat it for fear of the consequences. Most of the respondents sought relief from their symptoms through a combination of self-management and physiotherapy.

The impact of the injuries can be seen on the number of sick days taken. 10% (*n* = 6) of the trainees needed to leave work temporarily. A total of 20 days were lost due to work-related injuries during the last year, this may be underreported. We knew sickness abstinence can have an adverse impact on training and service delivery with financial implications [7]. This also affects the rest of the work force.

Work-related musculoskeletal injuries are more likely to be cumulative trauma disorders. Excessive and inappropriate body postures have been widely associated with high prevalence of WRMSIs more than other factors [29]. According to Vanwonterghem et al.’s [5] explanation about injury occurrence process as a result of cumulative exposure, an inevitable musculoskeletal injury is observed unless the body system has sufficient time for recovery. It can thus be suggested that adequate time should be provided for recovery to prevent recurrent injuries.

One of the limitations of this study is that the situations or health conditions might be over or underreported by the participants, depending on their personal views. However, some evidence showed that self-report gives similar results with experts’ examinations in the presence of musculoskeletal conditions among workers [30–32]. Another potential concern with survey studies is non-response bias because people with symptoms would be more likely to participate. However, the response rate for the present study was very high which would mostly eliminate this limitation. The participants for this study were limited by one special occupational group in one region. Although the current study is based on a small sample of participants, the combination of findings provides some degree of the magnitude of the problem among maternity professionals. We did not collect information on gender as we have a very small sample of male trainees in this region and it would not have been possible to carry out any meaningful correlative based in gender difference. Therefore, further research should be undertaken to investigate other aetiological factors. According to an international systematic review of 63 studies analysing the results of the interventions to reduce or prevent MSI [33], there is a moderate evidence that intervention strategies based on risk factor assessment were most likely to be effective. Once the risk factors are identified, the information would be useful in planning strategies to reduce or prevent the injuries.

Overall, there appears to be a lack of information on ergonomics and prevention strategies specific to obstetric work injuries. It is important to understand the importance of the ergonomics of obstetrics before we can develop prevention strategies that can be incorporated into training.

## Conclusion

The study was undertaken to find out the prevalence of work-related injuries and its detrimental effects. The most obvious finding to emerge from the analysis is that more than half of the obstetrics and gynaecology trainees experienced some degree of WRMSI that results in time off from work. Such injuries are underreported on incident reporting system. There is a lack of information on ergonomics and WRMSI prevention in obstetrics and gynaecology and further research is required to establish relevant aetiological factors related to work-related injury in the specialty so that the strategies can be developed based on the risk factors assessments to prevent such injuries.
